# Supplementary figures and images for: Baseline gene expression in subcutaneous adipose tissue predicts diet-induced weight loss in individuals with obesity
Source: PeerJ. 2023 Mar 24;11:e15100. doi: 10.7717/peerj.15100 (PMC10042157; doi:10.7717/peerj.15100)

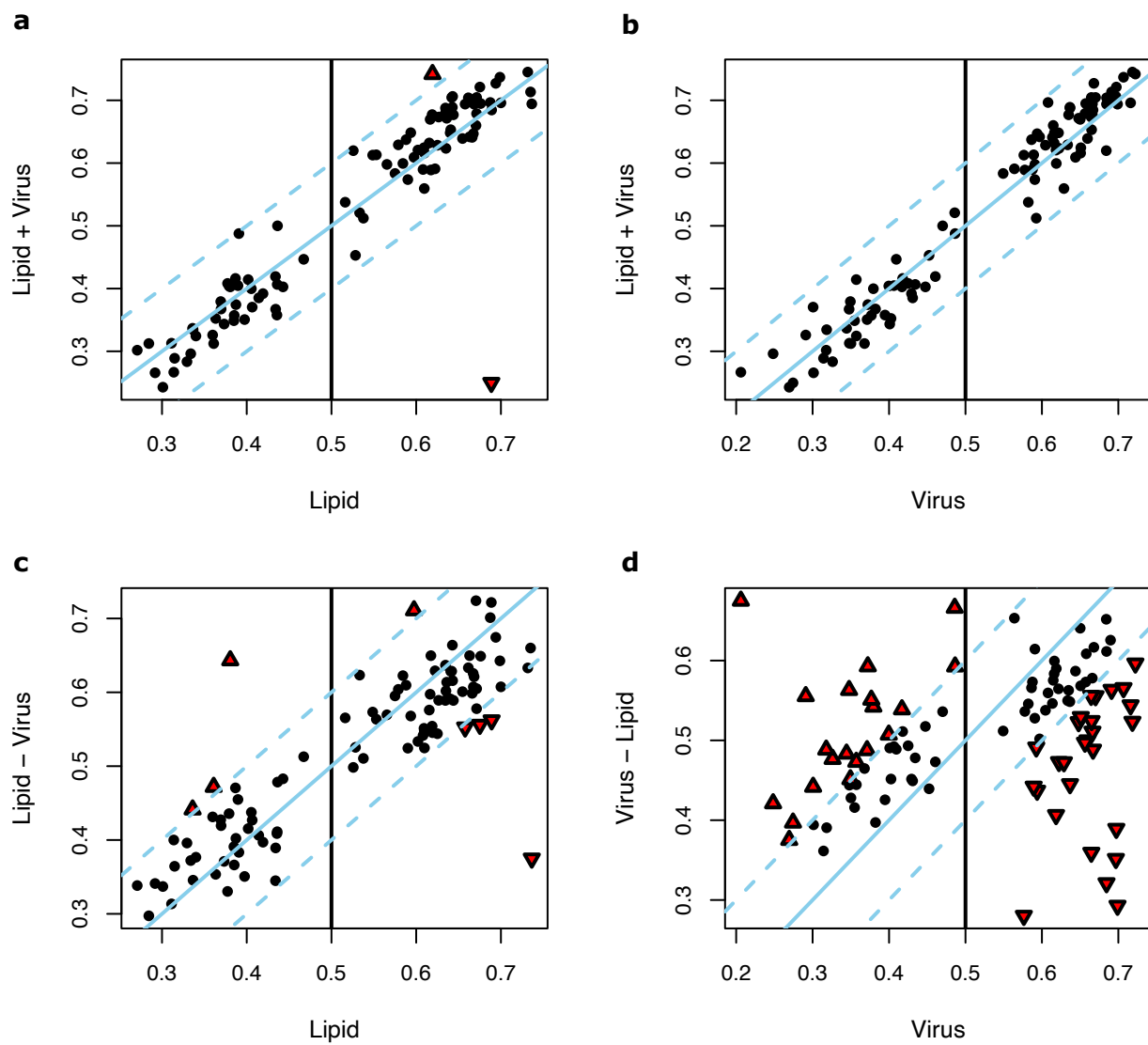

**Supplemental figure S1: Merging and subtracting parameters of Lipid and Virus models.**

Supplement: Supplemental Information 1 — (A and B) Scatter plots illustrating AUC measurements for weight loss prediction models based on the combined lipid metabolism and ‘response to virus’ genes vs AUC measurements of (A) Lipid models and (B) Virus models. (C) Scatter plots illustrating AUC measurements for weight loss prediction models based on lipid metabolism genes absent in Virus models, vs AUC measurements of Lipid models. (D) Scatter plots illustrating AUC measurements for weight loss prediction models based on ‘response to virus’ genes absent from Lipid models, vs. AUC measurements of Virus models. Dots or triangles indicate individual runs. The dots represent those runs where the AUC performance differences between the models that Y an X axis represent were less than 0.1. The threshold cutoffs (|Y−X| = 0.1) are shown with dashed lines. The solid line represents Y = X. The red triangles show the runs where the difference of the two models are significant (|Y−X| > 0.1). [file peerj-11-15100-s001.pdf]

**a**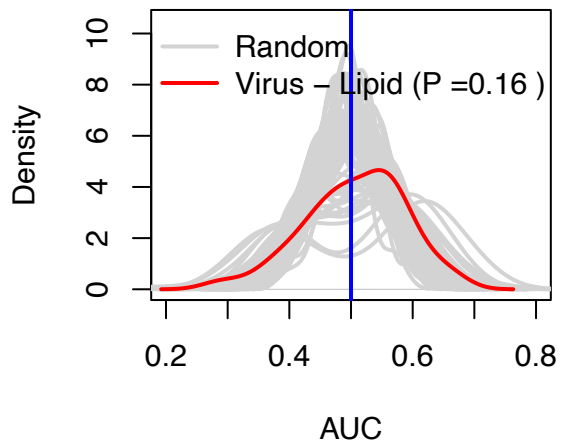**b**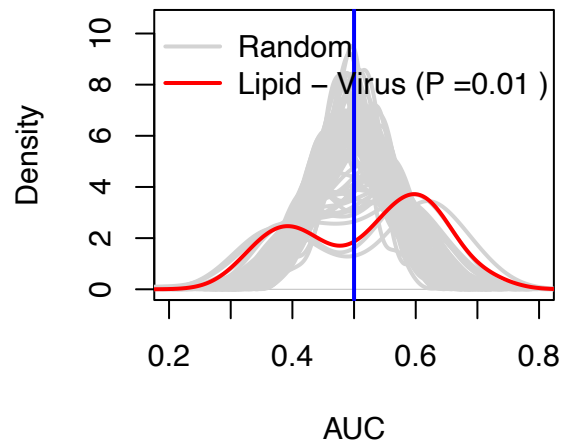

**Supplemental figure S2: Performance of Virus – Lipid and Lipid – Virus models.**

Supplement: Supplemental Information 2 — (A) Density plots illustrating the Area Under ROC Curve (AUC) measurements of models based on ‘response to virus’ genes absent in Lipid models (across the 100 cross-validation runs). (B) Density plots illustrating the Area Under ROC Curve (AUC) measurements of models based on lipid metabolism genes absent in Virus models (across the 100 cross-validation runs). The gray lines correspond to the AUC measurements for 100 prediction models constructed of randomly selected genes. The P value describes the probability that the models based on the randomly picked genes performs better than the Virus–Lipid or Lipid–Virus models. The vertical line at 0.5 AUC indicates the expected performance for random prediction. [file peerj-11-15100-s002.pdf]
