# Supplementary material for: Baseline gene expression in subcutaneous adipose tissue predicts diet-induced weight loss in individuals with obesity
Source: PeerJ. 2023 Mar 24;11:e15100. doi: 10.7717/peerj.15100 (PMC10042157; doi:10.7717/peerj.15100)
Supplement: Supplemental Information 3 — Density plot illustrating the Area Under ROC Curve (AUC) measurements of the prediction model that feature all 30 studied anthropometric and clinical factors as its input parameters, across the 100 cross-validation runs. The grey lines correspond to the AUC measurements for 100 prediction models constructed of randomly selected genes. The P-value describes the probability that the models based on the randomly picked genes performs better than the models that feature the anthropometric and clinical factors as their input parameters. The vertical line at 0.5 AUC indicates the expected performance for random prediction. [file peerj-11-15100-s003.pdf]

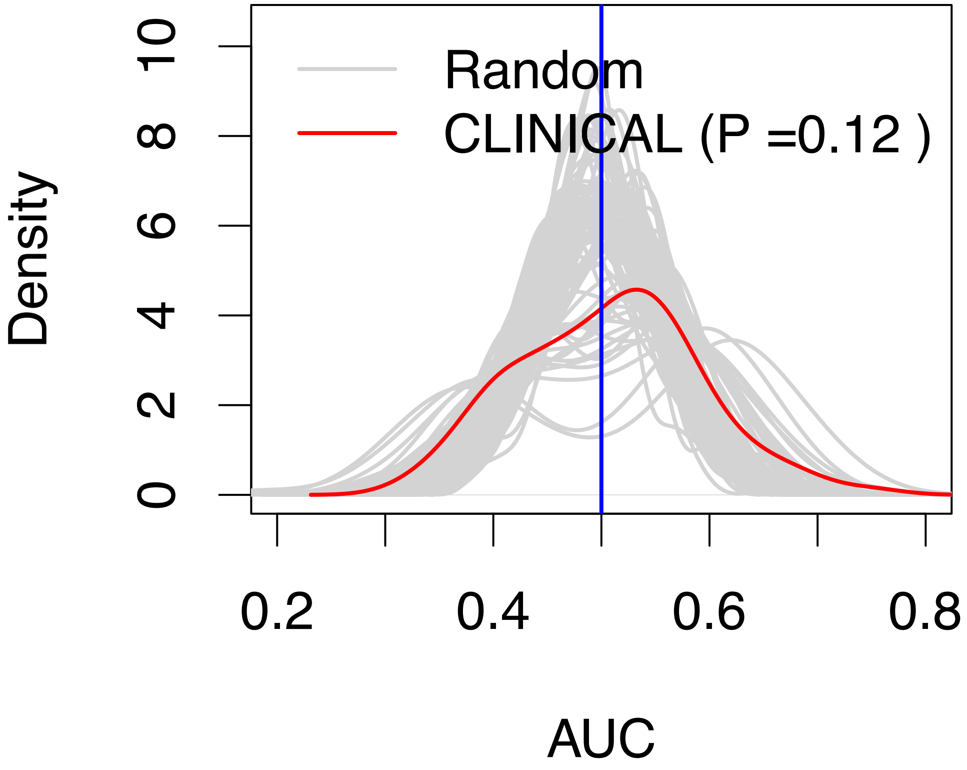

Supplemental figure S3: Performance of the prediction models based on baseline anthropometric and clinical factors.
