# Supplementary material for: Baseline gene expression in subcutaneous adipose tissue predicts diet-induced weight loss in individuals with obesity
Source: PeerJ. 2023 Mar 24;11:e15100. doi: 10.7717/peerj.15100 (PMC10042157; doi:10.7717/peerj.15100)
Supplement: Supplemental Information 4 — The P-values are the result of a paired t-test, comparing the performance of the corresponding prediction model to the performance of the Lipid prediction model. The models that improve the performance of the Lipid models significantly (Paired t-test, P < 0.05) are highlighted with magenta. [file peerj-11-15100-s004.docx]

**Supplemental Table S1. The performances of the Lipid prediction models when anthropometric and clinical factors were incorporated.** The P-values are the result of a paired t-test, comparing the performance of the corresponding prediction model to the performance of the Lipid prediction model. The models that improve the performance of the Lipid models significantly (Paired t-test, P < 0.05) are highlighted with magenta.

| **Prediction model** | **Median AUC** | **Mean AUC** | **Max AUC** | **p-value** |
| --- | --- | --- | --- | --- |
| Lipid | 0,582 | 0,528 | 0,736 |  |
| Lipid + BMI | 0,574 | 0,523 | 0,738 | 0,734 |
| Lipid + waist-hip ratio | 0,575 | 0,532 | 0,736 | 0,337 |
| Lipid + weight | 0,577 | 0,527 | 0,733 | 0,716 |
| Lipid + waist | 0,578 | 0,536 | 0,720 | 0,106 |
| Lipid + fat free mass | 0,526 | 0,513 | 0,767 | 0,935 |
| Lipid + fat mass | 0,515 | 0,507 | 0,750 | 0,970 |
| Lipid + fat percentage | 0,520 | 0,511 | 0,735 | 0,953 |
| Lipid + systolic blood pressure | 0,586 | 0,542 | 0,744 | 0,037 |
| Lipid + diastolic blood pressure | 0,582 | 0,534 | 0,730 | 0,244 |
| Lipid + cholesterol | 0,556 | 0,527 | 0,731 | 0,740 |
| Lipid + LDL cholesterol | 0,552 | 0,518 | 0,733 | 0,955 |
| Lipid + HDL cholesterol | 0,575 | 0,526 | 0,731 | 0,862 |
| Lipid + triglycerides | 0,562 | 0,527 | 0,726 | 0,672 |
| Lipid + free fatty acids | 0,517 | 0,511 | 0,739 | 0,942 |
| Lipid + c-reactive protein | 0,593 | 0,532 | 0,760 | 0,065 |
| Lipid + fibrinogen | 0,593 | 0,535 | 0,771 | 0,049 |
| Lipid + fructosamin | 0,587 | 0,528 | 0,733 | 0,492 |
| Lipid + factor VII | 0,594 | 0,535 | 0,754 | 0,069 |
| Lipid + adiponectin | 0,579 | 0,529 | 0,735 | 0,427 |
| Lipid + fasting glucose | 0,570 | 0,528 | 0,741 | 0,500 |
| Lipid + fasting insulin | 0,557 | 0,517 | 0,742 | 0,929 |
| Lipid + HOMA-IR | 0,577 | 0,528 | 0,755 | 0,506 |
| Lipid + Matsudas insulin sensitivity index | 0,554 | 0,528 | 0,742 | 0,513 |
| Lipid + insulinogenic index | 0,559 | 0,521 | 0,728 | 0,790 |
| Lipid + muscle insulin sensitivity index | 0,553 | 0,518 | 0,768 | 0,862 |
| Lipid + hepatic insulin resistance index | 0,537 | 0,517 | 0,763 | 0,865 |
| Lipid + adipose tissue insulin resistance index | 0,537 | 0,519 | 0,726 | 0,816 |
| Lipid + Baecke leisure index | 0,580 | 0,527 | 0,774 | 0,559 |
| Lipid + Baecke sports index | 0,573 | 0,524 | 0,741 | 0,700 |
| Lipid + Baecke work index | 0,585 | 0,524 | 0,773 | 0,673 |
| Lipid + all 30 above clinical factors | 0,516 | 0,508 | 0,731 | 0,893 |
