# Supplementary material for: Baseline gene expression in subcutaneous adipose tissue predicts diet-induced weight loss in individuals with obesity
Source: PeerJ. 2023 Mar 24;11:e15100. doi: 10.7717/peerj.15100 (PMC10042157; doi:10.7717/peerj.15100)
Supplement: Supplemental Information 5 — The P-values are the result of a paired t-test, comparing the performance of the corresponding prediction model to the performance of the Virus prediction model. The models that improve the performance of the Virus models significantly (Paired t-test, P < 0.05) are highlighted with magenta. [file peerj-11-15100-s005.docx]

**Supplemental Table S2. The performances of the Virus prediction models when anthropometric and clinical factors were incorporated.** The P-values are the result of a paired t-test, comparing the performance of the corresponding prediction model to the performance of the Virus prediction model. The models that improve the performance of the Virus models significantly (Paired t-test, P < 0.05) are highlighted with magenta.

| **Prediction model** | **Median AUC** | **Mean AUC** | **Max AUC** | **p-value** |
| --- | --- | --- | --- | --- |
| Virus | 0,588 | 0,525 | 0,722 |  |
| Virus + BMI | 0,579 | 0,531 | 0,787 | 0,220 |
| Virus + waist-hip ratio | 0,592 | 0,542 | 0,772 | 0,035 |
| Virus + weight | 0,576 | 0,524 | 0,724 | 0,736 |
| Virus + waist | 0,586 | 0,536 | 0,729 | 0,051 |
| Virus + fat free mass | 0,540 | 0,513 | 0,729 | 0,864 |
| Virus + fat mass | 0,536 | 0,515 | 0,733 | 0,861 |
| Virus + fat percentage | 0,539 | 0,518 | 0,750 | 0,787 |
| Virus + systolic blood pressure | 0,611 | 0,545 | 0,756 | 0,018 |
| Virus + diastolic blood pressure | 0,620 | 0,545 | 0,757 | 0,019 |
| Virus + cholesterol | 0,571 | 0,522 | 0,728 | 0,888 |
| Virus + LDL cholesterol | 0,572 | 0,525 | 0,733 | 0,461 |
| Virus + HDL cholesterol | 0,579 | 0,525 | 0,711 | 0,501 |
| Virus + triglycerides | 0,580 | 0,527 | 0,741 | 0,365 |
| Virus + free fatty acids | 0,540 | 0,520 | 0,718 | 0,679 |
| Virus + c-reactive protein | 0,581 | 0,529 | 0,751 | 0,112 |
| Virus + fibrinogen | 0,576 | 0,530 | 0,713 | 0,150 |
| Virus + fructosamin | 0,577 | 0,526 | 0,725 | 0,385 |
| Virus + factor VII | 0,581 | 0,530 | 0,761 | 0,127 |
| Virus + adiponectin | 0,577 | 0,535 | 0,777 | 0,078 |
| Virus + fasting glucose | 0,571 | 0,538 | 0,817 | 0,055 |
| Virus + fasting insulin | 0,569 | 0,531 | 0,733 | 0,221 |
| Virus + HOMA-IR | 0,571 | 0,534 | 0,800 | 0,169 |
| Virus + Matsudas insulin sensitivity index | 0,582 | 0,539 | 0,820 | 0,054 |
| Virus + insulinogenic index | 0,579 | 0,539 | 0,799 | 0,053 |
| Virus + muscle insulin sensitivity index | 0,566 | 0,531 | 0,738 | 0,263 |
| Virus + hepatic insulin resistance index | 0,551 | 0,530 | 0,799 | 0,305 |
| Virus + adipose tissue insulin resistance index | 0,538 | 0,520 | 0,716 | 0,698 |
| Virus + Baecke leisure index | 0,573 | 0,529 | 0,767 | 0,329 |
| Virus + Baecke sports index | 0,584 | 0,530 | 0,783 | 0,277 |
| Virus + Baecke work index | 0,582 | 0,527 | 0,779 | 0,408 |
| Virus + all 30 above clinical factors | 0,504 | 0,506 | 0,750 | 0,880 |
