# Supplementary material for: Baseline gene expression in subcutaneous adipose tissue predicts diet-induced weight loss in individuals with obesity
Source: PeerJ. 2023 Mar 24;11:e15100. doi: 10.7717/peerj.15100 (PMC10042157; doi:10.7717/peerj.15100)
Supplement: Supplemental Information 6 — The “P-value (Random)” column include the result of comparing the performance of the model to the models based on random selected genes. The column labelled with the “Paired t-test P-value” include the result of comparing the performance of the corresponding prediction model to the performance of the Lipid or the Virus prediction model. [file peerj-11-15100-s006.docx]

**Supplemental Table S3. The performances of the Lipid + Virus, Lipid – Virus and Virus - Lipid prediction models.** The "p-value (Random)" column include the result of comparing the performance of the model to the models based on random selected genes. The column labelled with the "Paired t-test p-value" include the result of comparing the performance of the corresponding prediction model to the performance of the Lipid or the Virus prediction model.

| **Prediction model** | **Median AUC** | **Mean AUC** | **Max AUC** | **p-value (Random)** | **Paired t-test p-value** |
| --- | --- | --- | --- | --- | --- |
| Lipid | 0,582 | 0,528 | 0,736 | < 0.01 |  |
| Virus | 0,588 | 0,525 | 0,722 | < 0.01 |  |
| Lipid + Virus | 0.590 | 0.529 | 0.745 | < 0.01 | 0.408 (Lipid), 0.105 (Virus) |
| Lipid - Virus | 0.550 | 0.519 | 0.724 | 0.01 | 0.107 (Lipid) |
| Virus - Lipid | 0.511 | 0.507 | 0.675 | 0.16 | 0.102 (Virus) |
|  |  |  |  |  |  |
